# A Mobile Health Intervention for Fetal Alcohol Spectrum Disorders (Families Moving Forward Connect): Development and Qualitative Evaluation of Design and Functionalities

**DOI:** 10.2196/14721

**Published:** 2020-04-06

**Authors:** Christie LM Petrenko, Jennifer Parr, Carson Kautz, Cristiano Tapparello, Heather Carmichael Olson

**Affiliations:** 1 Mt Hope Family Center University of Rochester Rochester, NY United States; 2 Department of Electrical and Computer Engineering University of Rochester Rochester, NY United States; 3 Department of Psychiatry and Behavioral Sciences School of Medicine University of Washington Seattle, NY United States; 4 Center for Child Health, Behavior, and Development Seattle Children’s Research Institute Seattle, WA United States

**Keywords:** fetal alcohol spectrum disorders, fetal alcohol syndrome, parenting, children, mobile health, mHealth, treatment

## Abstract

**Background:**

Fetal alcohol spectrum disorders (FASD) affect approximately 2% to 5% of the US population. However, most families are unable to access FASD-informed interventions. Barriers to care include the lack of a knowledgeable and skilled workforce and family-level barriers such as limited financial resources, inability to access childcare, and stigma. As a result, families often try peer-to-peer and self-help support strategies. However, they often take these strategies from disparate sources, which have quite variable intervention quality and empirical support.

**Objective:**

This study aimed to initiate systematic development and evaluation of a mobile health intervention *(app)* for caregivers raising children with FASD. Focus groups were conducted to elicit participant perspectives on app design and functionalities to inform further app development.

**Methods:**

The app, called *FMF Connect,* was derived from the scientifically validated *Families Moving Forward (FMF) Program,* a clinician-delivered behavioral consultation intervention. *FMF Connect* was intended for caregiver self-delivery and included five main components: (1) *Learning Modules,* (2) *Family Forum,* (3) *Library,* (4) *Notebook,* and (5) *Dashboard.* Focus group methods were used to solicit perspectives from diverse families during the early stages of app development. Questions were asked about interface design, relevance of components and content, and perceived barriers and facilitators of use. A total of 25 caregivers participated in 7 focus groups across 5 US cities. Data were analyzed thematically.

**Results:**

Focus group participants were generally enthusiastic about the app interface design and components. Four global positive impression themes emerged, including (1) ease of access, (2) how the app guides and organizes information, (3) connection to other users and information, and (4) ability to share some content with others. Themes arose not only in discussions relating to positive app features but also when participants were asked about motivators for app use. Participants related how these positive global themes could address some system-level barriers, such as limited access to services, feeling isolated, and increased advocacy needs related to the societal lack of FASD knowledge. Participants identified many positive features about individual app components and functionalities. They also communicated potential barriers to use and raised important concerns and considerations relating to several app components. These included recognizability of the app based on the logo, and the balance of following the planned intervention sequence versus obtaining immediate answers. Also mentioned were privacy and dynamics within the *Family Forum.*

**Conclusions:**

*FMF Connect* is a promising novel intervention with potential to reach many families in need and reduce significant barriers to care, resulting in a broader public health impact. Study findings will guide further app development both in terms of content and technological advances to optimize intervention effects. *FMF Connect* app development provides useful directions for other apps aimed at changing parenting practices.

## Introduction

### Background

Fetal alcohol spectrum disorders (FASD) affect an estimated 2% to 5% of the US general population [1] and occur at even higher rates in special populations, such as those served by child welfare and juvenile justice or in psychiatric care [2]. FASD are diagnosed when there is prenatal alcohol exposure and evidence of neurobehavioral impairment [3-5]. Additional symptoms may also be present. These include deficient brain growth or seizures, a pattern of characteristic facial features, and growth delays. Outcomes are variable and can be impacted by other factors such as maternal and fetal genetics, nutrition, immune functioning, and pre- and postnatal stress [6-8].

Unfortunately, research suggests that a majority of people affected by this condition are undiagnosed or misdiagnosed [9]. Children with FASD have high rates of health care and other service utilization [10,11], which should afford opportunities for detection and delivery of FASD-informed services and supports. However, significant system-level barriers exist that interfere with appropriate detection and service delivery [12,13]. One of the leading causes for system-level barriers is inadequate training in FASD provided to educators and health professionals. Surveys of trainees and professionals document that although many have heard about FASD, most do not feel competent to diagnose or effectively treat this condition [14-16]. As a result, there are very few providers in the community who regularly diagnose this condition or provide FASD-informed care. This problem is especially the case in rural or underserved communities.

Although an imperfect metric, an informal survey of resource directories from the National Organization on Fetal Alcohol Syndrome and their state affiliates show a median of 3 (range 0-13) diagnostic providers and 2 (range 0-86) mental health intervention services per state [17]. Clearly, existing resources are insufficient to meet the needs of this prevalent and often complex condition. It is also relevant to consider the demographics of families seeking FASD diagnostic and intervention services. Although services try to meet the needs of all family types, most families that receive FASD diagnostic services or participate in intervention research trials are foster parents, adoptive parents or relative caregivers (70%-98%) [18,19].

Families also experience many family-level barriers and stressors that adversely impact their ability to access FASD-informed care or even any care at all [13,20,21]. These include practical challenges such as finding appropriate childcare, financial strains, getting time off work, scheduling constraints, and juggling multiple demands and time-intensive services. Emotional stressors, such as feeling overwhelmed, isolated, and stigmatized, can impact the ability to access care. Parents raising children with FASD also report feeling discouraged by previous unsuccessful treatment outcomes, which can further reduce motivation to seek services.

### Potential of Mobile Health Interventions for Fetal Alcohol Spectrum Disorders

Since the 1980s, FASD parent support and advocacy groups have seen robust international growth in response to families’ urgent needs for help and support [22]. Families’ natural social support networks have been found helpful in the broader developmental disabilities literature [22,23], and caregivers of children with FASD often try peer-to-peer and self-help support strategies. With advances in technology and improvements in internet access, parents are more often obtaining information and social support from Web-based sources [24]. Social media has surpassed previously popular listserv and email formats for resource sharing and support [23]. Unfortunately, studies document multiple problems with the quality, consistency, and readability of Web-based information for parents of children with developmental disabilities [24].

Mobile health (mHealth) interventions are likely well suited to delivery of peer-to-peer and self-help support strategies for families raising children with FASD. Most adults have smartphones and regularly use apps in their daily lives [25,26]. mHealth interventions can potentially address many of the family- and system-level barriers currently interfering with access to FASD-informed care. One notable advantage of mHealth interventions is the ability to scale up for a large number of users. Developing mHealth interventions for FASD could also augment current programmatic efforts toward more traditional intervention dissemination and provider training, while capitalizing on the clear interest in and attempted use of self-help by caregivers raising children with FASD.

There are currently over 318,000 health-related apps available in app stores [25]. Remarkably, so far very few have been subjected to empirical study or are derived from evidence-based principles of behavior change [27-29]. Fortunately, evaluations of existing apps are becoming more common and clinical trials on mHealth interventions are increasing in number and quality [25,28]. Most apps evaluated to date have utilized health behavior theory constructs, such as self-monitoring and goal setting, and have received high user acceptability ratings [29]. Although sample sizes have been modest in intervention trials, preliminary evidence supports the potential for mHealth apps to accomplish behavior change, and ultimately improve outcomes across varied conditions [29,30].

### Evidence Base for Digital Parenting Interventions

Relatively few studies have explored the efficacy of mHealth interventions targeting parenting for preschool and school-aged children. However, a number of reviews and meta-analyses have summarized the growing evidence base for self-directed digital parenting interventions, which largely represent Web-based interventions [31-33]. The vast majority of self-directed interventions reviewed have been adapted from existing empirically validated interventions traditionally delivered by a clinician. In controlled trials, data suggest digital parenting interventions have similar or better retention (mean 84.8%) and adherence (mean 73.7% content completed) than in-person interventions [33]. Broadly, the evidence for digital parenting interventions is more consistent for interventions targeting externalizing behaviors than internalizing behaviors, given the very small number of such interventions focused on internalizing problems [33]. A meta-analysis of seven digital self-directed parenting interventions for children with externalizing behavior found overall small-medium effect sizes for child behavior (*d*=0.44), parent behavior (*d*=0.41), and parent confidence (*d*=0.36) [31]. Effects were larger for samples with clinically elevated behavior problems (*d*=0.61) than for nonclinical samples (*d*=0.21), and when interventions were interactive (*d*=0.82) versus noninteractive (*d*=0.36) [31].

Although smartphone technology is promising, few digital parenting interventions have capitalized on this method of delivery. Most interventions published to date have been developed for internet websites optimized for desktop or laptop computers. Yet, apps for smartphones and tablet computers have the advantage of greater ease of access for many parents during the course of a day. Importantly, integration of peer-to-peer support into mHealth and other digital parenting interventions could augment the power of these treatments. Integration could efficiently capitalize on benefits identified in previous research on social support. However, to date, the inclusion of peer-to-peer support within digital platforms has been surprisingly limited.

### Families Moving Forward Connect: A Novel Mobile Health Intervention for Caregivers Raising Children With Fetal Alcohol Spectrum Disorders

This study presents data from an initial evaluation of the design and planned functionalities of a novel mHealth intervention for caregivers raising children with FASD, called *Families Moving Forward (FMF) Connect*. *FMF Connect* is based on the caregiver-focused *FMF Program* developed by Olson and her research team at the Seattle Children’s Research Institute (SCRI)/University of Washington [19,22,34,35]. See Table 1 for the comparison of FMF and *FMF Connect*.

**Table 1:** Comparison of features of the standard Families Moving Forward (FMF) Program and FMF Connect mobile health intervention.

Features	Standard *FMF Program*	*FMF Connect*
Format	In-person; originally tested in families’ homes (but can also be delivered in-clinic or through telehealth)	Mobile health app
Target	Parents and caregivers of children (aged 3-12) with FASD or prenatal alcohol exposure	Parents and caregivers of children (aged 3-12) with FASD or prenatal alcohol exposure
Materials and delivery	Materials provided by specially trained mental health or child development provider	Materials are self-directed by the caregiver
Content division	14-17 sessions (includes core + optional material)	12 *Learning Modules* + optional material
Duration	90-min sessions, every other week (can be 60-min weekly sessions)	Self-directed by the caregiver
Clinical techniques	*Caregiver-focused:* Integration of psychoeducation and support, positive behavior support, cognitive behavioral strategies, advocacy education, and motivational interviewing	*Caregiver-focused:* Integration of psychoeducation and support, positive behavior support, cognitive behavioral strategies, advocacy education, and motivational interviewing
Key treatment processes	*Reframing, accommodations, brainstorming*	*Reframing, accommodations, brainstorming*
Key outcomes	Improve positive cognitive appraisal of child, improve parenting sense of competence, meet unmet family needs, and improve child adaptive function (and reduce problem behaviors)	Improve positive cognitive appraisal of child, improve parenting sense of competence, meet unmet family needs, and improve child adaptive function (and reduce problem behaviors)
Routine outcomes monitoring	Progress checklist completed at the start of each session to rate child behavior, self-care, and service barriers	Daily notifications to rate self-care and support, weekly notifications to rate child behavior
Social support	Support provided by a specialist, linkages to community or Web support groups	*Family Forum* for peer support integrated into app, moderator supported by training and consultation

#### Structure, Theoretical Framework, and Outcomes of the Families Moving Forward Program

The standard, therapist-led *FMF Program* was designed for parents and caregivers of children (aged 3-12 years) with FASD. The *FMF Program* is traditionally implemented in families’ homes every other week for 14 to 17 sessions, although in practice, it can also be delivered in clinic settings and in other patterns of session frequency or duration. The standard *FMF Program* was designed to fit with the highly diverse demographics of families raising children with FASD, including all family structures, and a wide range of socioeconomic status and caregiver racial and ethnic background.

The *FMF Program* is grounded in developmental systems theory, and is informed by research on parenting, developmental disabilities, family systems, and treatment of child behavior problems. It is designed to modify specific parenting attitudes and responses to children’s problem behaviors via integration of psychoeducation and support, positive behavior support, cognitive behavioral strategies, and motivational interviewing [19,22,34,35]. By helping caregivers interpret their children’s behavior from a neurodevelopmental perspective (called *reframing* in the standard *FMF Program*), it is theorized they will develop a more positive and realistic cognitive appraisal of the child, use more effective antecedent-based behavioral strategies to promote adaptive child functioning and decrease challenging behavior, and feel more efficacious in their role as a parent.

Among other findings, studies have documented generally medium to large intervention effects on caregiver knowledge, family needs met, parenting efficacy, reported improvement in self-care, and child behavior immediately posttreatment [19,36]. Effects on *reframing* and targeted parenting practices have been in the small to medium ranges [19,36]. Given how difficult are the lives of these children and families, improving the positive trajectory in any measurable way is a significant (and vital) aim.

#### Deriving the Families Moving Forward Connect Mobile Health Intervention

**Figure 1:**
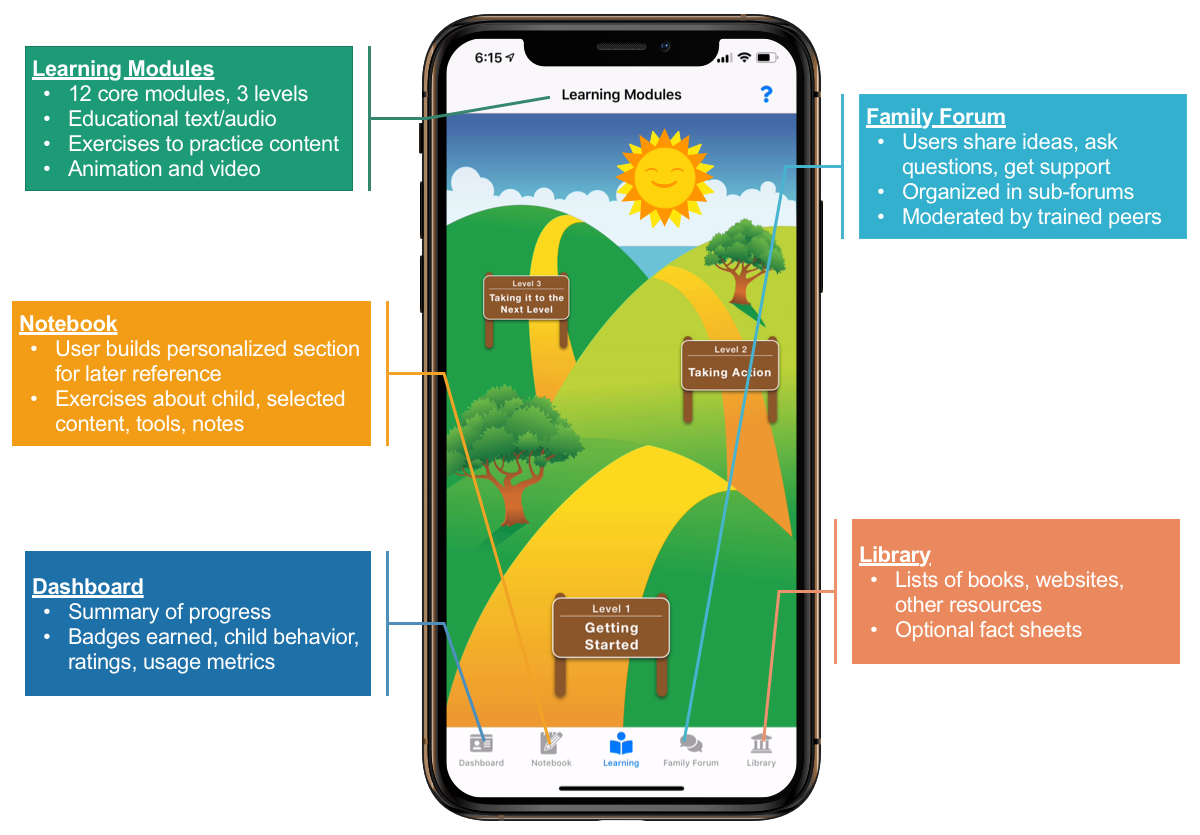
Illustration of the five main components of the Families Moving Forward (FMF) Connect mobile health intervention and their primary functionalities.

Consistent with the adaptation approach advocated by Card et al [37], the core components and underlying theory of the standard *FMF Program* were first identified. These components and theorized mechanisms of change were linked with technological features that correspond to how users interact with technology versus a literal adaptation to a new delivery mode [38]. This process was facilitated by the use of a backward design process [39], evaluation of behavior change techniques [40], and consideration of ethical principles [41]. See Figure 1 for an illustration of the *FMF Connect* components.

Psychoeducation, attitude change, and skill-building content from the standard *FMF Program* is distilled into brief learning modules. Standard *FMF Program* materials (short fact sheets, simple worksheets, and brief videos) lend themselves well to mHealth adaptation. Although much of the content is preserved, the flow of content delivery differs somewhat in *FMF Connect* to be more amenable to self-direction by caregivers.

Although much of the content translated well, potential mismatches between the original program and new delivery context were considered [37]. Several examples of this intensive process are given here. Difficult concepts introduced early in the standard *FMF Program* were simplified and moved later in the *FMF Connect* app flow because of the absence of clinician support. Since it is self-directed, *FMF Connect* was designed to rely much more heavily than does the standard *FMF Program* on video examples filmed with real families.

Derived from the standard *FMF Program* techniques, *FMF Connect* also prompts caregiver ratings of key outcomes (eg, child behavior, caregiver self-efficacy, and self-care) displayed on the app dashboard. These ratings capitalize on the confirmed benefits of *routine outcomes monitoring* for enhancing treatment effectiveness and tailoring intervention content [42].

Given the benefits of parent social support [22], an important innovation of *FMF Connect* is to integrate a peer-moderated *Family Forum* to aid in engaging families, promote implementation of new knowledge and skills, and provide secure online and high-quality support. The use of trained peer moderators promotes sustainability and surmounts workforce barriers, building on and enhancing what has naturally evolved in the *real world* of FASD self-help.

Other functionalities are also included, some of which were inspired by the standard *FMF Program*, and some unique to or transformed by the app format. These include a *Library* for additional optional content, a *Notebook* to organize completed exercises and tools, and weekly emails to engage users and highlight app features and tips.

### This Study

The relatively small, but quickly growing, evidence base for mHealth interventions is promising and offers direction and guidance. This study represents a crucial step in the systematic development of *FMF Connect* with a focus on app design and functionalities. Using rigorous qualitative methods, the app design and planned functionalities were presented to groups of caregivers of children with FASD in multiple cities across the United States. The integration of key stakeholder feedback early and iteratively throughout the development and evaluation process is aimed to facilitate the acceptability and utility of the intervention [43,44].

## Methods

### Study Design

The aim of this study was to elicit feedback about the design and components of the *FMF Connect* mHealth intervention from targeted users: caregivers of children with FASD. Qualitative methodology is well suited to the aims of this study. Focus group methods were specifically chosen to elicit in-depth discussion among caregivers on aspects such as interface design, ease of use, relevance of components and content, and barriers and facilitators of use.

### Recruitment

Given that the app is designed for use by families across the United States, caregiver perspectives were elicited across various geographical regions. Caregivers were eligible for the study if they were over the age of 18 years and a primary caregiver of a child (aged 3-17 years) with an FASD. Diagnosis was based on caregiver report, although most participants were recruited through well-established diagnostic clinics. Although *FMF Connect* is designed for caregivers of children aged 3 to 12 years, caregivers of adolescents (aged 13-17 years) were also included. These caregivers have the advantage of being able to reflect on their experiences parenting their child across the full age range targeted by the app. They can also offer a broader perspective on the types of features and content that would be helpful. Caregivers who had previously completed the standard *FMF Program* were also included in this study. They could reflect on previous lived experience learning and applying the content of the *FMF Program*. In addition, they could offer important insights on what it might be like to learn this content in self-directed manner through *FMF Connect*.

Caregivers were recruited through multiple mechanisms. These included existing FASD research registries, provider referrals, targeted flyers in parent support groups or Web newsletters, and conferences. Several principal investigators within the Collaborative Initiative on FASD (CIFASD) also offered to help with recruitment and logistics for holding focus groups at their sites.

A total of 25 caregivers participated in 7 focus groups across 5 US cities from December 2017 to June 2018. Each focus group included 3 to 4 caregivers. Focus groups were held in Rochester, NY (3); Atlanta, GA (1); Minneapolis, MN (1); San Diego, CA (1); and Los Angeles, CA (1).

The University of Rochester Institutional Review Board reviewed and approved all study procedures. Participants provided written informed consent before enrollment in the study.

### Procedures

All focus group interviews were conducted in a private meeting room at each site. In preparation, participants completed a brief demographic questionnaire, including some metrics of their comfort with technology and smartphone usage.

A consistent research team conducted all focus group interviews. The first author, who is a clinical psychologist and researcher with 15 years of experience in the field of FASD, was the lead moderator of all focus groups. She has 7 years of experience using qualitative research methods and multiple published studies with this population [12,21,45]. The fourth author is a faculty member in computer engineering. He led the demonstrations of design mock-ups and app prototypes in focus groups. The second author is a doctoral student in counseling and counselor education and took detailed observational notes during all groups. No personnel apart from participants and researchers were present during the interviews.

Each focus group session began with research team introductions, a statement about the purpose of the focus group, and discussion of ground rules and expectations. To reduce positive response bias, participants were explicitly encouraged to share any concerns or negative feedback during focus groups. The research team emphasized they would rather hear these concerns during development when changes could more easily be made than later once the app was widely disseminated.

At the start of discussion, participants were provided with a handout giving brief bullet point descriptions about each component of the *FMF Connect* app. The research team, then, showed participants mock-ups or prototypes of the app design (see Multimedia Appendix 1). They, then, elicited participant discussion component by component, following a semistructured interview guide. After reviewing individual app components, participants were asked about general impressions of the app interface and perceived motivators and barriers to use. The interview guide included open-ended questions (eg, “What do you think about the *Family Forum*?” “What might you improve or do differently?” “What do you think about the look and feel of the app?” and “What would motivate you to use an app like this?”) and additional probes to elicit more in-depth responses, when needed.

After each focus group, the research team reflected on key themes discussed and further refined the interview guide. Novel ideas or considerations raised by participants during earlier groups were also posed to later groups for discussion. Examples included gradual access to subforums, seeking immediate answers versus learning module progression, and privacy concerns. All interviews were audio recorded with participants’ consent. Participants were provided a US $20 cash incentive for their participation.

### Data Analysis

Audio recordings from focus groups (average duration=91 min, range 79-109 min) were transcribed verbatim by the research team and rechecked for accuracy by the second author. Detailed observational notes recorded by the second author during focus groups were integrated within transcripts. For example, observational data included nonverbal gestures (eg, head nods), distracted or nonengaged behaviors (eg, looking at phone), and affect and tone of voice. Data were, then, imported into Atlas.ti (version 8.3.1, ATLAS.ti Scientific Software Development GmbH, Berlin, Germany) for coding and analysis.

A thematic analysis was undertaken to understand participants’ perspectives on the app design and functionalities. The purpose was to inform further app development. Thematic analysis focuses on identifying patterns or themes within the data [46,47]. Three research team members conducted primary analyses. This included the first two authors (both involved in data collection), with the additional perspective of a graduate student in clinical psychology not involved in collecting original data (third author).

Consistent with the approach advocated by Miles et al [47], research team members each familiarized themselves with the data, iteratively reviewed each transcript, and independently assigned initial codes. The team, then, came together and discussed, operationalized, and refined each code. Transcripts were, then, recoded. Codes were refined through further discussion and consensus. The research team examined interrelationships and networks among codes, then built and refined the analytic model. Participant matrices [47] were utilized to examine variance in themes across participants and several key demographic features (eg, previous participation in FMF). Participant demographic variables were also imported into Atlas.ti and code co-occurrence tables were examined to assist with this process.

## Results

### Sample Demographics

Table 2 provides participant demographics. All participants were either adoptive parents or relatives of the child. In all, 80% (20/25) of the sample was female, with caregiver age ranging from 35 to 73 years. Although a wide income range was represented in the sample, over half of the sample had annual family incomes over $75,000. The sample resided mainly in suburban areas. Mean child age was 8.1 years. Intentionally, 40% (10/25) of caregivers had previously received the standard FMF Program. Although 40% (10/25) of participants rated themselves as *very comfortable* with technology, a wide range of perceived comfort is represented in this sample.

**Table 2:** Participant demographics.

Sample characteristics			Value
**Caregiver type, n (%)**			
	Adoptive parent	18 (72)	
	Grandparent	5 (20)	
	Other relative	2 (8)	
**Caregiver gender, n (%)**			
	Female	20 (80)	
	Male	5 (20)	
**Caregiver age (years)**			
	Mean (SD)	51.36 (10.29)	
	Range	35-73	
**Caregiver race/ethnicity^a^, n (%)**			
	White	23 (92)	
	Black/African American	2 (8)	
	Native American/Alaskan Native	2 (8)	
	Hispanic/Latinx	4 (16)	
**Caregiver education, n (%)**			
	High school diploma/ General Education Development	4 (16)	
	Some college/associates degree	7 (28)	
	Bachelor’s degree	6 (24)	
	Master’s degree	6 (24)	
	Doctoral/professional degree	2 (8)	
**Estimated annual family income (US $), n (%)**			
	Less than 25,000	1 (4)	
	25,000-34,999	4 (16)	
	35,000-49,999	1 (4)	
	50,000-74,999	4 (16)	
	75,000-99,999	3 (12)	
	Over 100,000	9 (36)	
	Did not answer	3 (12)	
**Type of Community, n (%)**			
	Rural	3 (12)	
	Suburban	20 (80)	
	Urban	2 (8)	
**Age of child(ren) (years), n=40^b^**			
	Mean (SD)	8.1 (3.96)	
	Range	1-17	
**Previous receipt of FMF^c^, n (%)**			
	Yes	10 (40)	
	No	15 (60)	
**Comfort with technology**			
	Mean (SD)	5.20 (1.92)	
	1 *I find it very difficult*, n (%)	1 (4)	
	2, n (%)	2 (8)	
	3, n (%)	2 (8)	
	4, n (%)	4 (16)	
	5, n (%)	3 (12)	
	6, n (%)	3 (12)	
	7 *I am very comfortable*, n (%)	10 (40)	

^a^Nonexclusive categories. No participants identified as Asian, Native Hawaiian/Pacific Islander, or Other.

^b^Several parents also had younger children with a fetal alcohol spectrum disorder, in addition to a child within the study age range.

^c^FMF: Families Moving Forward.

Participant matrices were examined across themes to assess for differences based on demographic characteristics. Overall, themes identified were fairly consistent across focus groups and did not generally differ based on participant demographics. Caregivers who had previously received the standard *FMF Program* occasionally referenced the program when discussing a positive feature of FMF Connect. However, themes did not generally differ (with one exception noted in the Organizing/Guiding theme section) relative to those who had not completed the program. In addition, participants with older children or adolescents were more likely to raise the need for interventions for adolescents and adults. They otherwise communicated similar themes as did participants with younger children.

### Global Impressions

Participants were generally enthusiastic about the app and had positive global impressions. Four global impression themes emerged in analysis, which include (1) ease of access or accessibility of the app; (2) the app’s ability to guide and organize key information; (3) how the app connects users with information, resources, and other caregivers; and (4) the ability to share information with people outside the app. These themes arose not only in discussions relating to app positive features but also when participants were asked about motivators for using the app and unprompted discussions about system-level barriers (see later section on this topic: *How Families Moving Forward Connect Addresses System-Level Barriers*). Evidence for each of these themes is presented in the following sections. Table 3 is a participant matrix illustrating the high level of agreement among participants on these themes.

**Table 3:** Participant matrix for global impression themes. Codes in theme cells indicate when participants gave one or more extended utterances (EU) or simple agreement (SA) to comments related to each theme.

Focus group and participant identification number		Previous FMF^a^	Ease of access	Guiding/organizing	Connection	Share with others
**Rochester 1**						
	FG001	Yes	SA^b^	EU^c^	EU	EU
	FG002	No	EU	EU	EU	EU
	FG003	Yes	SA	—^d^	SA	—
	FG004	Yes	EU	EU	EU	EU
**Rochester 2**						
	FG005	Yes	EU	EU	EU	EU
	FG006^e^	Yes	SA	EU	EU	EU
	FG007^e^	Yes	SA	SA	EU	SA
	FG008	Yes	EU	EU	EU	SA
**Atlanta**						
	FG009	No	SA	—	SA	SA
	FG010	No	EU	—	EU	EU
	FG011	No	—	—	EU	EU
**Minneapolis**						
	FG012^e^	Yes	EU	EU	EU	EU
	FG013	No	EU	EU	EU	SA
	FG014	No	EU	EU	EU	EU
**Rochester 3**						
	FG015	Yes	EU	EU	EU	EU
	FG016	Yes	EU	SA	EU	EU
	FG017	No	EU	EU	—	EU
**San Diego**						
	FG018^e^	No	EU	—	SA	EU
	FG019^e^	No	EU	—	EU	SA
	FG020	No	SA	—	EU	EU
	FG021	No	SA	—	EU	SA
**Los Angeles**						
	FG022	No	EU	—	EU	SA
	FG023	No	EU	SA	—	SA
	FG024	No	EU	EU	EU	EU
	FG025	No	SA	EU	—	SA

^a^FMF: standard Families Moving Forward Program.

^b^SA: Simple agreement, defined as at least one single word (eg, *yes*, *I agree*) or nonverbal nod relating to the theme.

^c^EU: Expanded utterance, defined as at least one multiple word phrase or sentence(s) reflecting the theme.

^d^Dashes indicate a participant did not provide a clear nonverbal or verbal response relating to this theme.

^e^Caregiver of an adolescent (aged 13-17 years).

#### Ease of Access

This theme includes two aspects. First, participants referenced app accessibility as a positive feature. They thought the accessibility of the app would allow more people to obtain needed information and strategies for raising a child with an FASD. For example, one participant said enthusiastically:

I think it’s awesome because so many more people are going to have access to the Families Moving Forward.

FG024

Second, participants spoke of the benefits of having information in one place they could easily access. One caregiver said:

What I like about the app is you’re not digging through all the papers you were dealing with before [whole group nods yes].

FG005

Participants described most needing this information in emotional or stressful moments. The app provides an easy way for parents to meet their needs in such moments. This idea is highlighted by the following quote:

...It sounds easy enough for me to navigate through…which is very, very good because… sometimes you’re overwhelmed and you’re frustrated and you wanna look at something quickly…I can then navigate through that with no problem and get the information.

FG015

#### Guiding and Organizing

This theme was discussed in five of the seven focus groups. The two groups where this theme was not discussed had no participants with previous FMF experience. Participants referenced the fact that the app guides users through step by step learning, starting with the basics and then building on that foundational knowledge. For example, a participant said:

Oh I like it so far, very much. And I’m looking at the different content and, I mean definitely it looks like you have built from one thing to the next to help us, … it’s good to conquer what came before, before you go on to the next one.

FG017

Parents appreciated that the app offers a single place to aggregate and organize information about their child or children. The organization of this information allows parents to track patterns and changes in their children, which can often be difficult and time consuming. For example, one participant said:

That [tracking behavior] would be so amazing because I know when I go to the different doctors, or psychiatrists, or neurologists and they ask and I’m like, last week, last month, last day…it’s all just one…That would be amazing… you’re just able to pull that up and have it right there [FG025 nodding yes] or have a quick way to say like “oh, it’s happening again."

FG024

#### Connection

Participants were enthusiastic about how the app connects users with key information, resources, strategies, and other caregivers in the Family Forum. The connection provided by the app was commonly regarded by participants as a motivator to use the app. This is revealed in the following interaction among three participants:

Well, I think the forum is going to keep me coming back too.

FG008

Yes, it’s that social media part of it, that’s going to keep me coming back.

FG006

And I think having access to…information on different services that are available.

FG007

Participants also emphasized the ability to share ideas with other users as a positive feature of the app. They were especially interested in having subforums within the Family Forum where they could connect with other users in their geographical area and share information on local resources. For example, a participant said:

If there’s a way to identify other members that are in your geographical area… We’re also always sharing resources. Like, who gets it? I’m so tired of signing up for therapy and the therapist doesn’t have a clue what [FASD] is.

FG012

#### Share With Others

Participants recognized that many people who work with their child will not be using the app. They realized providers and school staff will not always have access to or be aware of key information presented in the app. Because of this, parents really liked the option to download fact sheets from the *Library* to share with providers, especially teachers. One parent shared:

And, I just think the idea of being able to pull little pieces of it here and there out and helping others understand is very exciting.

FG017

Similarly, another parent said:

It would be great if there was a way to have that as one of the resources, a document you could share with teachers. Um, and maybe even something attached to the child specifically, based on your observations.

FG014

This theme generated a lot of enthusiasm in groups, especially in Atlanta and San Diego. As discussed below in the *Identified Remaining Needs* section, participants offered additional content and feature suggestions related to this theme.

### Individual Components

**Figure 2:**
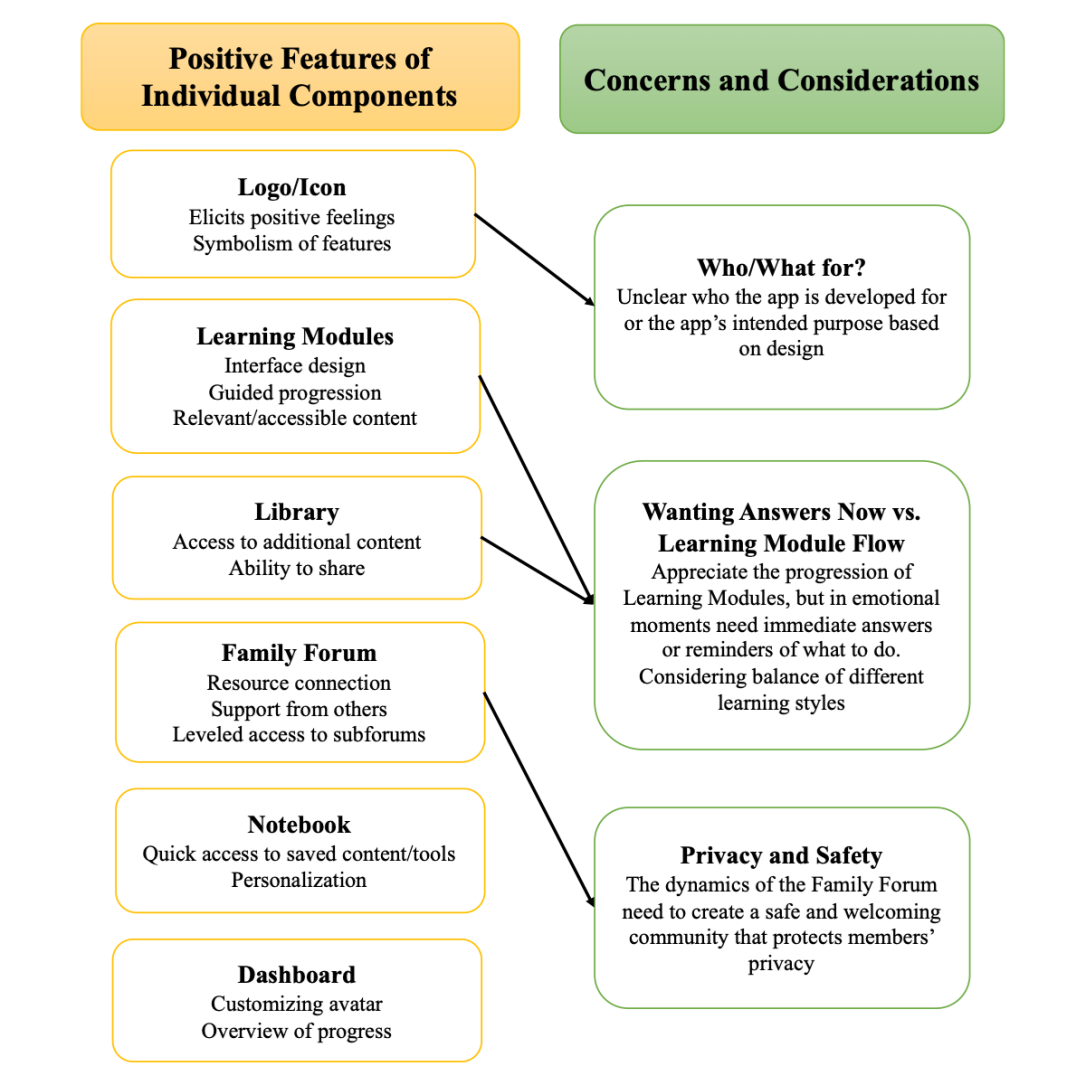
Primary themes relating to the main individual components of the Families Moving Forward (FMF) Connect mobile health intervention. Themes fell into two main categories: (1) positive features and (2) additional considerations and concerns. The considerations and concerns tended to come up in relation to specific components.

Themes related to individual components fell into two categories: (1) positive features based on high enthusiasm and participant discussion, and (2) additional considerations or topics with which participants grappled. In reviewing the data, concerns and considerations tended to come up in relation to specific components. Opinions were mixed or evolved over the course of discussion. A number of constructive suggestions were offered about how to respond to these considerations. In the following sections, both positive features and concerns or considerations will be discussed in the context of individual components. Figure 2 illustrates these graphically.

#### Families Moving Forward Connect Logo and Icon

**Figure 3:**
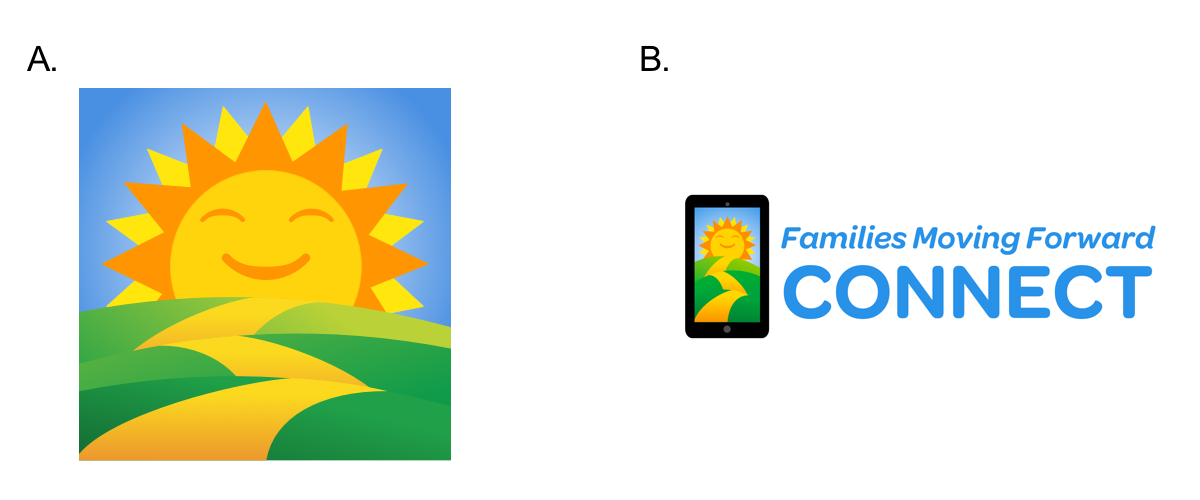
Part A of this figure is the icon shown to participants in focus groups. For the latter focus groups, this was shown on a phone simulator. Part B of the figure is the Families Moving Forward (FMF) Connect logo presented to participants.

Generally, the logo and icon did not generate a great deal of enthusiasm or engaged discussion (see Figure 3 for illustration). However, some consistent themes emerged. The logo and icon elicited positive feelings such as feeling hopeful, optimistic, and happy. Participants also commented on the symbolism of features. For example, one participant stated:

I do like the crooked path, because it doesn’t look overly simplistic to me. It does convey that this is a process and that there are steps to it, so I really like that.

FG014

The concern that was raised by participants for this component is a theme labeled *Who/What for?* Participants noted that it was unclear who exactly this app was developed for from the first glance at the logo or icon. Participants stated that the logo and icon felt somewhat childish and that they may mistake the app as a game for children. The following quote from a participant represents this dilemma

It does look aimed towards children, so I wouldn’t know it was the right one.

FG002

Although the concern about recognizability was raised, participants felt that with time and exposure to the app, the logo and icon would become recognizable and they would associate the logo and icon with the app.

The face on the sun was identified as a key feature by multiple participants contributing to the childish nature of the logo and icon. For example, a participant shared that the icon “pushes this state of euphoria that none of us parents probably have ever experienced. So that piece to me is not reality. And then the sun makes it feel very childish” (FG018). Suggested changes mainly had to do with changing the face of the sun or trying to add features into the logo to make it more clearly related to FASD or brain-based disabilities.

#### Learning Modules and Library Components

**Figure 4:**
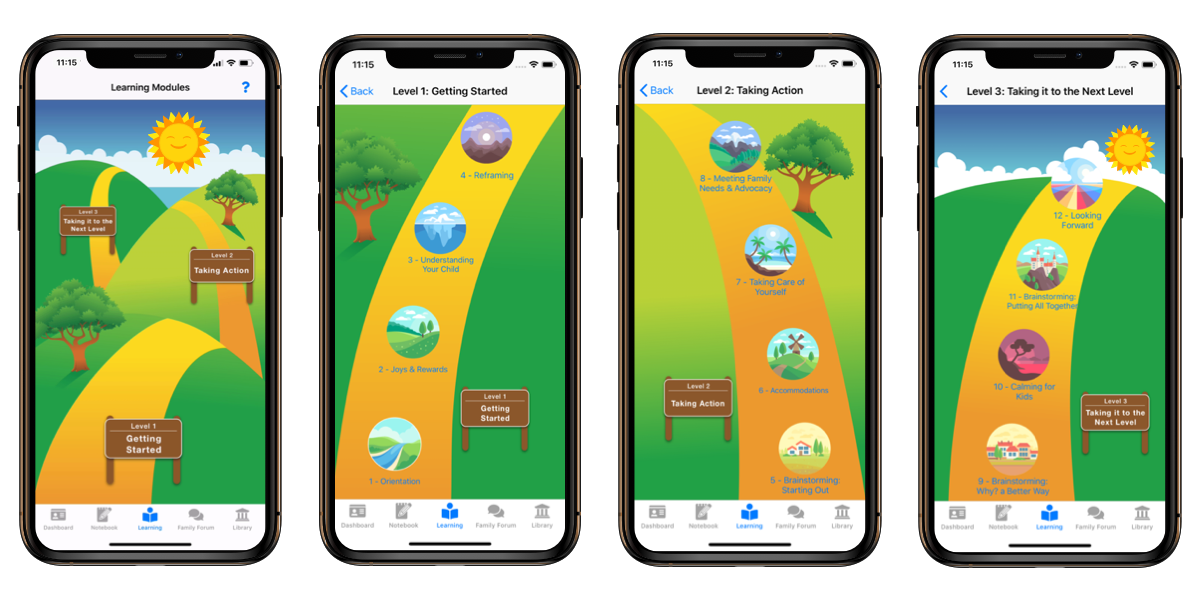
Screenshots of the Learning Modules interface. The first screen is the Learning Modules home page that shows the three levels: (1) Getting Started, (2) Taking Action, and (3) Taking it to the Next Level. The next three screens show the individual modules within each of these three levels.

Participants spoke positively of the interface design of the *Learning Modules*. In particular, they enjoyed the individual icons for each of the learning modules (see Figure 4 for the screenshots of *Learning Module* home screens). The primary critique relating to the design interface was that progressing through the *Learning Modules* from the bottom to the top of the screen was, at first, somewhat counterintuitive.

Participants appreciated the guided nature and step-wise aspect of the learning content, allowing users to build on knowledge and skills from previous modules. They also thought the content areas were relevant. They especially liked having all of the information accessible in one app. This quote represents a caregiver expressing how valuable she felt this content would have been, early on, in raising her child:

I would’ve loved to have had those at the very beginning [whole group looks at FG019 and nods yes]…to have learned…what is FASD. Just like she was saying [gesturing towards FG018], there’s so many places to go learn about it but if you’re in one central place that is done by professors and people in the know, that would be awesome to be able to do that.

FG019

Participants often spoke of the *Library* in relation to the *Learning Modules*. They were positive about being able to extend their learning in the *Library* beyond the standard content offered in the *Learning Modules*. In addition, participants valued that the *Library* allows them to download fact sheets to share with other people in their children’s lives.

Regarding the *Learning Modules* and the *Library*, participants grappled with one main concern. This was how to balance the guided progression of learning content with the acknowledged fact that people want content immediately available to navigate at their own pace and direction. Most participants recognized the importance in progressing through the learning content in a step-wise fashion. For example, one caregiver said:

You want the modules to work. You know, you want when they get to the end they’ve accomplished a great thing that will really be a resource for them to go back. It never helps when you’re trying to do something that’s nonfiction to jump to the end of the book or to start in the middle. [whole group nodding yes].

FG017

However, the point was raised that different people have various learning styles and may want access to all the material at once. For example, a participant commented:

I understand that you want sequence and building upon the knowledge that you’ve learned, but I still want more available in the library ahead of time.

FG025

This was a lively discussion during many of the focus groups. Participants predicted they would want answers immediately during times when they feel stressed or emotional, or when their children are struggling. The following quote provides an example of a participant who wrestled with understanding the importance of the *Learning Module* progression, while recognizing there are times that require immediate answers:

I could see myself, “I want this one, I need the calming strategies right now!” You know? So I understand that moving parents along on a continuum is important and getting them to do the education is important, but hopefully you’re also going to find an audience of people that are fairly adept with some of this stuff and they really want to make good use out of specific components as well.

FG012

On the more extreme end of this continuum, one participant was clear that having to follow the *Learning Module* progression to get the information she wanted would be a barrier to her using the app:

If I can’t get a resource within a reasonable amount of time and energy then apparently I don’t need that resource… I wouldn’t take the route [indicating learning module route] just because I don’t know if getting to the end is going to give me enough of a benefit to be invested in it. So, I probably personally would not use it.

FG018

#### Family Forum

Participants were very excited about being able to share resources and ideas with other caregivers in the *Family Forum*. They were also excited about having a space to connect with and gain support from other caregivers. For example, one participant stated:

...because I love the fact that if someone had a great idea – not necessarily that it’d work for my kid but it’s a possibility…I love the idea of exchanging ideas [FG006 nodding yes emphatically].

FG005

Another participant spoke to the value of getting support from other caregivers who understand their experience:

...You’re in this one forum because other parents have been there or are going through it and it’s not that you’re wanting, ‘oh, it’ll be okay,’ but you need a spot to be able to vent.

FG010

An idea was raised by participants in the first focus group to open certain subforums after the completion of particular *Learning Modules*. They suggested that by opening subforums based on module completion, users would be able to know they are talking with other parents that have the same level of familiarity with the content. That would mean that users could ask for support or advice based on more advanced concepts. This idea was proposed to each subsequent group and was viewed positively.

The biggest concern consistently raised in relation to the *Family Forum* was the importance of ensuring that forum dynamics fostered a safe and supportive community. Protecting one’s privacy was often mentioned in these discussions. Participants frequently referenced their experiences in Facebook groups when discussing forum dynamics and safety or privacy concerns. Indeed, forum dynamics were described as a key factor for using the app. One participant stated:

One thing with Facebook, it keeps you coming back because there’s interaction, there’s back and forth, it’s not just me and my app and...That’s where the forum is going to be important. But the forum’s got to be a safe place. [whole group nods emphatically].

FG001

In contrast, several participants indicated they would be less likely to use the *Family Forum*. Two participants (FG022 and FG023) stated that they were not social media users and did not think they would use this feature much. Another participant (FG012) thought the features of the *Family Forum* were duplicative of benefits she already receives through existing Facebook groups, but noted other parents might find it helpful.

Participants felt that the presence of trained peer moderators would address the concern of privacy and safety. They also felt it would be beneficial to have clear guidelines to help users provide and seek out productive support. Some participants also talked about a protection strategy of choosing to share different levels of information, depending on the nature of the group. For example, one participant stated:

On Facebook there’s many different FASD groups and for me personally, how I handle them is I talk very generally on the more national ones… In our local FASD group, I share very intimately…But that’s because it’s a local, it’s monitored, and we screen.

FG004

#### Notebook and Dashboard

The final two components of the app are the *Notebook* and the *Dashboard*. These components did not directly relate to any of the major concerns or considerations brought up by participants and were generally referenced in a positive manner. Participants appreciated that the *Notebook* provided quick access to saved content, as well as to various tools in the app. In addition, they liked that the *Notebook* could provide a personalized section and history of their child. For example, one participant stated:

...so that way you can make your own quick reference guide, you know what I mean, within your notebook....So that would keep me coming back.

FG005

The *Dashboard* was in the early stages of development when shown to focus groups; therefore, there was limited discussion surrounding this component. Generally, people liked that they could create and customize an avatar and that the dashboard would provide a quick overview of *Learning Module* progress.

### Perceived Barriers to App Use

Participants described potential barriers to using *FMF Connect*. Possible barriers fell into four main themes. The first theme involves technological aspects such as the app loading slowly or crashing. For example, a participant (FG024) said that she would not use the app, “if it was ‘buggy’ and it was hard to like get to the things that I needed – it just like repetitively didn’t work.” The second theme involves whether information will be presented in ways that are overly complex and involve too much scrolling or navigation between screens. A participant described:

For me, scrolling I don’t do scrolling. It gives me nausea [FG006 nods enthusiastically]. So, I would rather have chunks of information and then click next.

FG008

The third theme includes forum dynamics such as low user activity, negative tones, and overly judgmental posts or comments. For example, a participant said:

If people were very negative, very judgmental, I would probably not use it.

FG003

The fourth theme raised time and money as other potential barriers. Participants discussed how precious time is when raising children. Therefore, to make it worth their time, they felt the app should be accessible and easy to move through. For example, a participant said:

Obviously cost would be a factor. That if it was too costly, it wouldn’t be worth my time. ...But again, I’m never opposed to paying...a reasonable amount for something that I’m getting value from.

FG020

### How Families Moving Forward Connect Addresses System-Level Barriers

Although not specifically queried by the moderator, participants often raised their experience of system-level barriers, resulting in some lively (and at times emotional) discussions based on shared experiences. The most common system-level barriers raised were (1) limited access to services, (2) feeling isolated, and (3) having to advocate for their children because of providers’ and teachers’ lack of knowledge about FASD. These barriers are especially notable given the relatively high educational and financial demographics of many families in this sample.

Most of the positive global impression themes were described by participants as helping to meet some needs relating to these barriers. For example, participants spoke passionately about not being able to access services and having very limited access to FASD-informed care. Participants felt the app addressed this barrier by providing a service that was easy to access and offered connections to other parents who may provide suggestions for additional local services. For example, one participant who had just completed the standard *FMF Program* after being on a waitlist for several months said:

So now you wouldn’t have to wait so long… I had hiccups in my life that made it where I had to not go for a little while. Where if I had an app I could have done it at home.

FG016

Next, participants commonly spoke of feeling isolated in their experience of raising children with FASD. They commented that the support and connection provided in the app would help to alleviate or reduce that feeling of isolation. One participant said

And I think it’s a very often isolating experience and...I really like...the idea that you can connect with other parents and learn from them.

FG013

Finally, participants felt the unmet need for advocacy support due to lack of knowledge among providers and teachers would be addressed. This is because users can easily share information from the app with others as a means of advocating for their child in different settings. These advocacy efforts were especially important to participants in reference to school settings. The following quote is fairly representative of participants’ discussions of school advocacy and how the app might support their efforts:

Can you have handouts that we can give to teachers? Like this is FASD…this is how their brain works sometimes…that kind of stuff that’s easy to give a teacher…like an overview of whoever you might be working with. Like a quick, simple this is mostly what you’ll see in my kid.

FG024

### Identified Remaining Needs

Although the app addresses some of the system barriers faced by families, participants identified additional needs that remain. Related to the theme of ease of access, caregivers asked whether app content could be accessed across multiple platforms, such as tablets or internet browsers on computers. They identified that using multiple platforms would make it easier to engage with different app components or different settings. For example, a participant said:

I would really like to access it on both [whole group nods yes]. You know for that fast access on the phone but, if I really want to spend time in the app I’d really much prefer it to be on the computer.

FG008

Participants also felt very strongly that they should have continued access to the app, even after finishing all *Learning Modules* or a certain length of time had passed. Continued access would allow caregivers to maintain connections with other users in the family forum. Continued access also allowed them to refresh knowledge and skills by reviewing key content. For example, one participant said

I definitely think that our children...they change so much. [Whole group nodding yes]...what their needs are, what medications they’re on, changes a lot...And so I think being able to go back as if it was one of your favorite books.

FG017

A number of families were raising multiple children with FASD, each of whom had quite different needs. Participants wanted a way to consider the needs of multiple children in the app. For example, a participant emphasized the differences between his two daughters with FASD and stated:

If you don’t address both of them [in the app] you’re going to be lost as a parent.

FG011

Although participants appreciated the connection provided from the *Family Forum*, they would like it taken a step further by including a resource directory of FASD-informed providers and community resources. Participants also wanted additional features in the app to be used by their children, often related to calming strategies.

Finally, parents requested the development of adjunct or companion apps to aid in advocacy efforts with providers, teachers, respite workers, and other family members. Some participants were raising children older than those targeted for the app. Those parents highlighted the need for apps to be created to support adolescents, adults, and their caregivers.

## Discussion

### Principal Findings

This study represents an important step in the systematic development and evaluation of the *FMF Connect* mHealth intervention for caregivers raising children with FASD. Inclusion of key stakeholder feedback early in the app development process is a major strength of this process [43,44]. App-based interventions have the advantage of scalability and can potentially reach many in need and reduce significant barriers to care [12,48]. *FMF Connect* is one of the few parenting interventions of its kind. It is based on an intervention tailored for its diverse target population and is the first self-directed mHealth intervention for FASD.

Results from this study revealed that participants were largely enthusiastic about the app’s initial design and functionalities. The positive global impression themes identified by participants (ease of access, guiding and organizing, connection, and share with others) are consistent with the functions for which mHealth apps are well suited [29,49]. Participants related how these positive global themes could address some of the system-level barriers they encounter. Examples include limited access to services, feeling isolated, and high advocacy needs related to a lack of knowledge about FASD. The positive global themes were also primary factors identified by participants that would motivate them to use the app.

Participants evaluated many positive features about individual app components and functionalities. Yet, they also identified potential barriers to using the app, raising some important concerns and considerations relating to several app components. This knowledge will inform further refinements and evaluation of the app. For example, the suggestion by participants to open subforums based on module completion has already been implemented in a beta-testing version of the app. Similarly, feedback relating to behavior tracking and icon design has been taken into account in app refinement. Further, features supporting improved *Family Forum* dynamics have been added. This iterative and systematic approach to app development makes it more likely that the app will be acceptable and effective for families.

### Limitations

FMF Connect is being developed for the US population. Although efforts were made to recruit a diverse sample of caregivers raising children with FASD, some subgroups are not well represented in the study sample. Multiple geographic areas of the United States were sampled. Given resource limitations and logistics, however, coverage did not reach all regions. Most notably, the study did not enroll any biological parents, despite multiple recruitment efforts designed to engage this important subgroup. It is possible that many biological parents may not be comfortable in group research settings because of stigma or other factors. Future studies could include alternate data collection methods, such as individual interviews, or identify other relationship-building strategies to engage this group. It is important to remember that birth parents comprised about 15% overall of the standard *FMF Program* research participants, so the original material was designed to be sensitive and useful with that subgroup. Videoconferencing may also work to include members of other geographic areas or underrepresented subgroups. The next stage of the evaluation of the *FMF Connect* app beta-testing will (1) integrate focus group and interview data collection methods and (2) explore videoconferencing as a method to increase sample diversity.

Although themes were similar across groups, the size of each focus group was smaller than anticipated. A total of 4 to 12 participants were scheduled for each group but, unfortunately, because of inclement weather, illness, and other unanticipated scheduling conflicts, participant turnout was lower than expected. A smaller group size did not seem to negatively impact the flow of discussion, but larger groups might have resulted in additional themes or different patterns of results.

Potential for possible biases should also be considered. The research team is developing the app, which could elicit a positive response bias from participants. In addition, some participants knew the moderator from other Rochester area FASD services, and other participants were recruited through CIFASD investigators at other sites. These participants may have come to focus groups with previous positive associations with the research team or their colleagues. Although the moderator encouraged negative feedback during focus groups and participants gave a range of critical and constructive feedback, it is impossible to completely rule out this source of bias.

Participants with strong, vocal opinions or different experiences (eg, previous FMF involvement) could also potentially influence themes in focus groups. The moderator made efforts to elicit feedback from all participants and seek differing opinions. Findings from this study do not suggest past FMF involvement influenced data, with the exception of possibly the guiding/organizing theme. Selection bias is also possible. Participants who had a favorable view of apps may have been more interested in participating in the study.

### Broader Applicability of Findings

Findings from this study may inform other mHealth apps with families raising children with FASD or other special needs. This study engaged key stakeholders in the early development process of the *FMF Connect* intervention. Such engagement has been a critical process in the adaptation of other evidence-based interventions to a self-directed digital format [50]. This study showed the utility of stakeholder feedback, which yielded specific ideas that could be implemented immediately or used to guide further exploration.

For *FMF Connect*, caregivers thought the learning module content was relevant and gradually built on foundational knowledge. This input suggests that adaptations to the standard *FMF Program* sequence for the *FMF Connect* app make sense and are acceptable to stakeholders. However, participants grappled with retaining the step-wise progression of the intervention versus obtaining immediate answers and advice. Although several ideas were suggested, no clear solution was identified. This will be a key consideration to explore in next steps of prototype refinement with iterative feedback from stakeholders.

Participants were enthusiastic about being able to share ideas and connect with other caregivers in the *Family Forum*. They felt that connecting with other parents could reduce the feelings of isolation and help address barriers of finding and accessing services for their children and family. These findings are consistent with qualitative research assessing in-person and online peer support for parents of children with special needs. Findings from this research literature reveal the assets of shared experiences, mutual support, encouragement, and knowledge sharing [51,52]. Positive outcomes commonly described include themes of improved coping and assurance about child management strategies, less isolation, and ability to support other families. In this study, participants tempered their excitement about the *Family Forum* with concerns about privacy and safety. These concerns were often based on their past experiences with social media such as Facebook. These concerns have also been raised in other studies [53]. The attention to fostering a safe, nonjudgmental, and welcoming environment was stressed. Having clear guidelines and peer moderators were viewed as positive protections in the *Family Forum*. These findings are informative for other mHealth interventions incorporating social media that are targeted to families of children with special needs.

Participants in this study placed positive emphasis on the accessibility and organization of content. The fact that content would be evidence based was viewed favorably. Previous reviews of Web-based information geared toward parents raising children with developmental disabilities document problems with the quality, consistency, and readability of information [24]. Participants in this study described gathering information from many different sources and sometimes struggling to find what they needed. They liked that the app would include evidence-based information all in one place. They were also enthusiastic about the *Notebook* component as a way to organize and individualize content for their child. These results suggest that deriving mHealth interventions from existing evidence-based treatments may be particularly well received by users, especially when apps tailor and personalize information.

Previous research has documented that self-directed digital interventions derived from well-studied therapist-led parenting programs are effective in improving child and parenting outcomes [31-33]. Effect sizes are larger for studies with clinical samples and interventions that use interactive content and formats to engage users [31]. In the field of FASD, two known studies have utilized Web-based intervention formats. Kable et al [54] found that a self-directed brief Web-based intervention for families raising children with FASD had multiple outcomes similar to a therapist-led workshop. The Strongest Families intervention, which integrates weekly Web-based content and coaching telephone calls with a trained coach, is also being tested with caregivers of children with FASD [55]. Although analysis is underway from the larger randomized controlled trial, early usability data with a small subsample found the website was easy to navigate and that content was written at a level that was understandable to families [56]. During early development, some challenges were identified with caregivers learning how to use the interface initially, having too much scrolling, and specificity of content to FASD, which were improved in subsequent testing. This body of research speaks to the potential of the *FMF Connect* mHealth app and the benefits of rigorous, systematic research. Much of the format, behavior change principles, and content of *FMF Connect* could be quite relevant for other parenting apps, especially those targeting parents of children with special needs.

### Conclusions

*FMF Connect* builds on a solid foundation of empirical research on a tailored intervention designed for families raising children with FASD [19,36]. Capitalizing on the promise of the field of mHealth, *FMF Connect* has the potential to reach many families in need and reduce significant barriers to care. This can result in broader public health impact. This study’s findings will guide further app development both in terms of content and technological advances to optimize intervention effects. Next steps will involve the completion of initial programming, iterative small-scale beta-testing and refinement, and larger feasibility testing. A large-scale randomized controlled trial is, then, planned to evaluate the efficacy with respect to caregiver and child outcomes. This rigorous development process can be an example in the field of mHealth and for the future of parenting interventions.

## Appendix

Multimedia Appendix 1 Mock-ups of the Families Moving Forward (FMF) Connect interface design shown to participants in focus groups. Screen mock-ups were shown to participants using an interactive interface in Invision or initial prototype (iOS). Components shown include: app icon, Dashboard, Learning Modules, Family Forum, Library, and Notebook.

## Abbreviations

CIFASD: Collaborative Initiative on Fetal Alcohol Spectrum Disorder
FASD: fetal alcohol spectrum disorder
FMF: Families Moving Forward
mHealth: mobile health
NIAAA: National Institute on Alcohol Abuse and Alcoholism
SCRI: Seattle Children’s Research Institute
