# Supplementary figures and images for: A Mobile Health Intervention for Fetal Alcohol Spectrum Disorders (Families Moving Forward Connect): Development and Qualitative Evaluation of Design and Functionalities
Source: JMIR Mhealth Uhealth. 2020 Apr 6;8(4):e14721. doi: 10.2196/14721 (PMC7171567; doi:10.2196/14721)

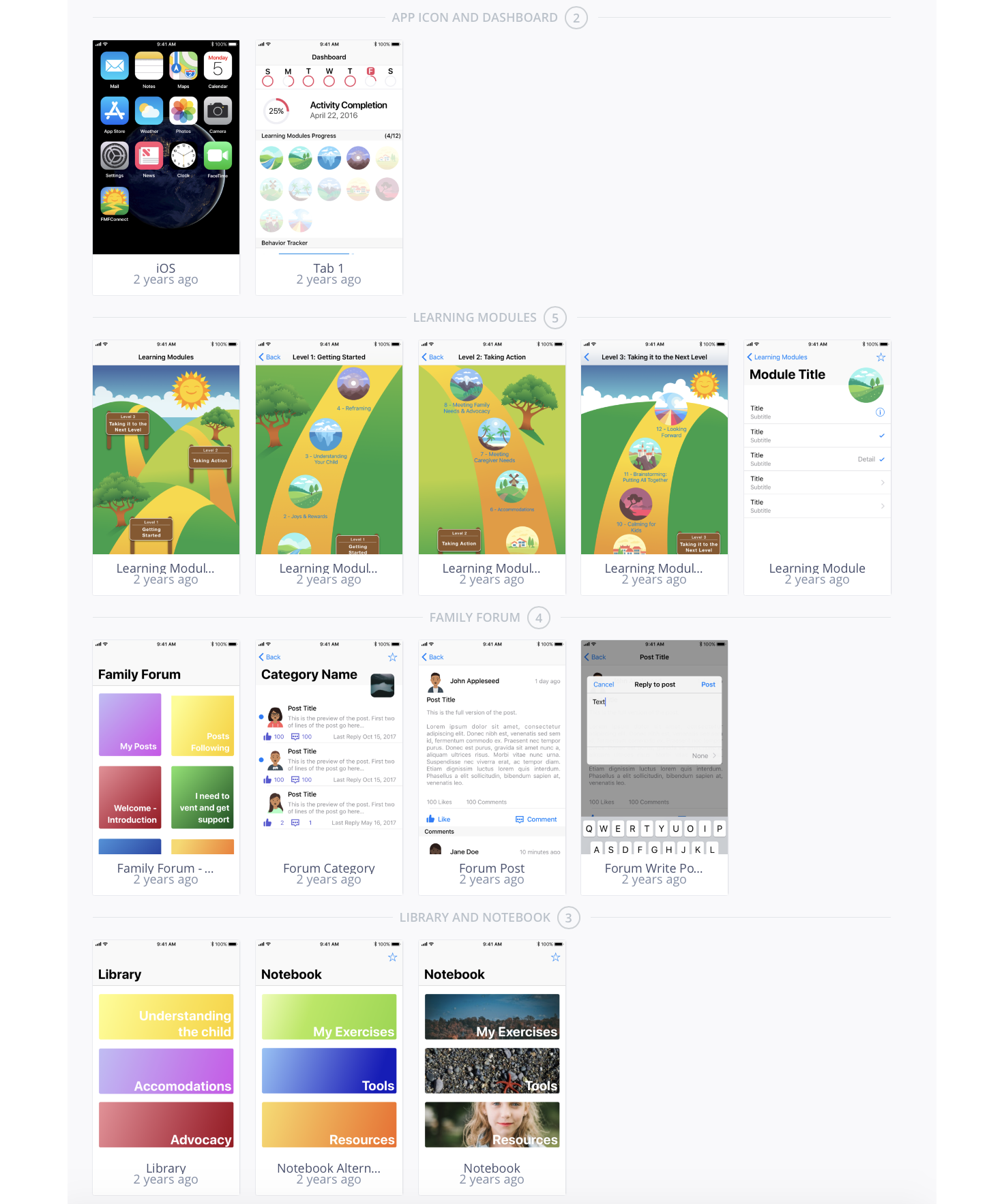

Supplement: Multimedia Appendix 1 [file mhealth_v8i4e14721_app1.png]
